# Impact of *Haemophilus influenzae* type b combination vaccination on asthma symptoms and pneumonia in 5-year-old children in rural Bangladesh: a longitudinal study and comparison with a previous cross-sectional study

**DOI:** 10.1186/s12931-021-01629-8

**Published:** 2021-02-03

**Authors:** Haruko Takeuchi, S. M. Tafsir Hasan, Khalequ Zaman, Sayaka Takanashi, Samar Kumar Hore, Sultana Yeasmin, Shaikh Meshbahuddin Ahmad, Md Jahangir Alam, Masamine Jimba, Tsutomu Iwata, Md Alfazal Khan

**Affiliations:** 1 Department of Community and Global Health, Graduate School of Medicine, The University of Tokyo, 7-3-1 Hongo, Bunkyo City, Tokyo 113-0033 Japan; 2 Nutrition and Clinical Services Division, International Centre for Diarrhoeal Disease Research, Bangladesh (icddr,b), 68, Shaheed Tajuddin Ahmed Sarani, Mohakhali, Dhaka 1212, Bangladesh; 3 Infectious Diseases Division, icddr,b, 68, Shaheed Tajuddin Ahmed Sarani, Mohakhali, Dhaka 1212, Bangladesh; 4 Department of Developmental Medical Sciences, Graduate School of Medicine, The University of Tokyo, 7-3-1 Hongo, Bunkyo City, Tokyo 113-0033 Japan; 5 Infectious Disease Surveillance Center, National Institute of Infectious Diseases, 1-23-1 Toyama, Shinjuku City, Tokyo 162-8640 Japan; 6 Organization for Population Health Environment & Nutrition, Adilpur Shastitala, Taltala Kheyaghat Road, Abhaynagar, Jashore 7460, Bangladesh; 7 Department of Biochemistry and Microbiology, North South University, Bashundhara, Dhaka 1212, Bangladesh; 8 Department of Microbiology, Biomedicine Discovery Institute, Monash University, Clayton, VIC 3800 Australia; 9 The Graduate School of Humanities and Life Sciences, Tokyo Kasei University, 1-18-1 Kaga, Itabashi City, Tokyo 173-8602 Japan

**Keywords:** Asthma, Bangladesh, Child, *Haemophilus influenzae* type b vaccine, Administration and dosage, Pneumonia, Protective factor

## Abstract

**Background:**

Although the prevalence of bronchial asthma has been increasing worldwide since the 1970′s, the prevalence among 5-year-old children was significantly lower in 2016 than in 2001 in rural Bangladesh. We aimed to determine whether the *Haemophilus influenzae* type b (Hib) combination vaccination (without booster) started in 2009 contributed to this decrease.

**Methods:**

A case–control study was conducted among 1658 randomly selected 5-year-old children from Matlab, Bangladesh. Data on wheezing were collected using the International Study of Asthma and Allergies in Childhood questionnaire. The vaccination data were collected from the records of the Matlab Health and Demographic Surveillance System, while data on pneumonia were obtained from the clinical records of Matlab Hospital. Adjusted odds ratios (aORs) were calculated for the risk for wheezing. The reduction rate was calculated to determine the impact of the vaccination on pneumonia history between the present study and our previous study conducted in 2001 by using the following formula: (percentage of pneumonia cases in 2001 − percentage of pneumonia cases in 2016)/(percentage of pneumonia cases in 2001) times 100 (%).

**Results:**

Hib combination vaccination was a protecting factor against wheezing (aOR: 0.50; *p* = 0.010), while pneumonia at 1, 2, 3–4 years of age were risk factors for wheezing (aOR: 2.86, 3.19, 2.86; *p* = 0.046, 0.030, 0.030, respectively). The history of pneumonia was significantly lower in the 2016 study participants than those in 2001 both in the overall cohort and the wheezing group (paired t-test: *p* = 0.012, *p* < 0.001, respectively). Whereas the history of pneumonia decreased when the children grew older in the 2001 overall cohort, it peaked at the age of 2 years in 2016 wheezing group. The reduction rate decreased when children grew older in both the overall cohort and the wheezing group, however, it decreased faster in the wheezing group.

**Conclusions:**

Hib combination vaccination was a protective factor against wheezing in 0-year-old children. However, the effects of vaccination might have attenuated at the ages of 1–4 years, because no booster dose was administered. The addition of a booster dose might further decrease the prevalence of asthma and wheezing.

## Background

Bronchial asthma is characterized by chronic inflammation and hyper-responsiveness of the airway and is one of the most common chronic diseases of childhood [1]. The established risk factors for asthma are atopy and respiratory tract infections [2, 3]. The contribution of environmental factors to the development of asthma first came to light when the prevalence of asthma markedly increased in the 1970′s in industrialized and high-income countries [4–6], as it appeared unlikely that genetic factors alone would have contributed to this sudden increase. Indeed, subsequent epidemiological studies did find a large variation in the worldwide prevalence of asthma, and confirmed that asthma was more common among children in urban, industrial and affluent areas than among children in rural areas [7, 8]. More recently, however, a significant decline in the prevalence of wheezing was found among 5-year-old children in rural Bangladesh, from 16.2% in 2001 to 8.7% in 2016 [9, 10], despite the obvious improvement in socio-economic status (SES) during this period. A nationwide cross-sectional survey in 1999 reported a prevalence rate of 7.3% for asthma among children aged 5–14 years [11], while a study in 2000 documented a prevalence rate of 9.1% for “current wheezing” among school children aged 6–7 years [12]. In 2001, the asthma prevalence among 5-year-old children was documented as 16% [9], and in 2003, an even higher prevalence of current wheezing, nearly 20%, was recorded among 5-year-old children in rural Bangladesh [13]. These findings imply that some specific factor must have inhibited the high prevalence of wheezing or asthma, although the studies were not based on the same methods.

Meanwhile, there had been a lot of improvements in the area which influenced child health including the care of respiratory infections, which is a strong risk factor for wheezing and asthma. The mortality from respiratory tract infections greatly decreased due to various and rigorous measures. These include antibiotic use by the community health research workers (CHRWs), Vitamin A distribution, improved hospital care, improved referral, improved nutritional status, etc. *Pneumoccocal* vaccine was also introduced in 2015, which the participants of the 2016 study were not administered, though.

In 2009, the Expanded *Program* on Immunization (EPI) of Bangladesh introduced three doses of *Haemophilus* influenzae type b (Hib) combination vaccine, Shan5™ (SHANTHA BIOTECHNICS PRIVATE LIMITED, Telangana, India), in the form of pentavalent vaccine that included Diphtheria, Tetanus and Pertussis (DTP) vaccine, hepatitis B vaccine and Hib vaccine without a booster dose. Besides, in 2004, Bangladesh started the national deworming program, which was developed to administer anti-helminthic drugs to children aged 24–59 months, with another program (also initiated in 2004) that delivered the same drugs to primary school children to eliminate soil-transmitted helminthiasis.

In rural Bangladesh in 2001, 26% of childhood wheezing was attributable to anti-*Ascaris* IgE [14], and 16% of childhood wheezing was attributable to the history of pneumonia during young childhood [14], which was the major causes of death of young children in Bangladesh [15]. The prevalence of wheezing seems to have decreased concurrently with the decrease in the prevalence of *Ascaris* infection, implying that the implementation of the deworming program caused the decrease [10]. However, *Ascaris* infection itself was not found to be a risk factor for wheezing [10].

While the Hib combination vaccine has been reported to prevent invasive infectious diseases by Hib, such as meningitis [16], it also had an impact on pneumonia, which proved to be a strong risk factor for asthma and wheezing [9, 14], and has even been reported to lead to herd protection in Bangladesh [17]. Since few studies have reported the association of Hib vaccination with childhood asthma and wheezing, the present study aimed to determine if the introduction of Hib combination vaccination and various measures for pneumonia in Bangladesh contributed to the reduction in childhood asthma and wheezing, through pneumonia prevention.

## Methods

### The aim, design and setting of the study

The aim of the present study is to determine if Hib vaccination contributed to the decrease of asthma and wheezing through pneumonia prevention.

We conducted this case–control study on the risk of Hib combination vaccination and history of pneumonia for asthma symptoms among 5-year-old children in Matlab. We also compared the results of the present study with those of our previous study (described below) to determine whether Hib combination vaccination contributed to decrease pneumonia prevalence [9], the result of which was partially unpublished. We finally compared death from respiratory infections including pneumonia among children under 5 years of age in the area before and after the introduction of Hib combination vaccination using reports of health and demographic surveillance system (HDSS) run by icddr,b, formerly the International Centre for Diarrhoeal Disease Research, Bangladesh (ICDDR,B), to determine the impact of Hib combination vaccination on pneumonia related mortality [18–24].

Matlab is a low-lying riverine area, where the principal occupations are farming and fishing. Since 1966, the HDSS, which consists of regular cross-sectional censuses and the longitudinal registration of vital events, has been maintained in the area by the icddr,b [24]. The population of the HDSS area, which encompasses 142 villages, was approximately 220,000 in 2001 and 230,000 in 2014 when the present study was initially planned. A maternal and child health, and family-planning program serve approximately half of the population of the HDSS area (the icddr,b service area, which covers 67 villages). A record-keeping system (RKS) records all instances of immunization in the service area.

The present study was conducted from December 2015 to October 2016, and planned to include 1800 children aged 5 years who were randomly selected from all 67 villages of the HDSS service area. A total of 1658 children ultimately participated in the study. The International Study of Asthma and Allergies in Childhood questionnaire was used to identify wheezing. One hundred and forty-five children had experienced wheezing during the previous 12 months and 1513 children had not. The 145 children who experienced wheezing during the previous 12 months were placed in the “wheezing” group and 1513 children who did not experience wheezing during the previous 12 months were placed in the “non-wheezing” group. The participants of this study were able to receive Hib combination vaccine since it was introduced in the area in 2009. We compared these children with the participants of our former study conducted in 2001. The total population of our 2001 study consisted of 1705 children, who were all children aged 5 years from 51 villages that were themselves randomly selected from the service area [9]. Data on deaths from respiratory infections including pneumonia were obtained from the Matlab HDSS Scientific Reports published by the icddr,b [18–24].

The study protocol was approved by the Ethical Review Committee of the icddr,b (PR-15054). The Ethics Committee of Tokyo Kasei University (Sayama H27-09), and The University of Tokyo (11018 and 2020180NI) approved the study. The protocol of our 2001 study was approved by the Ethical Review Committee of the International Centre for Diarrhoeal Disease Research, Bangladesh (2000-038). As the studies involved human subjects, the ethical principles of the Declaration of Helsinki were followed. Written informed consent was obtained from the legal guardians of all participants.

### Field data collection

The procedures used for data collection in the present study have been described elsewhere [10]. In brief, trained local field-research assistants visited the homes of the children and collected information using a semi-structured, pre-tested questionnaire adopted from the International Study of Asthma and Allergies in Childhood questionnaire [25]. Wheezing was defined as any episode of wheezing or whistling in the chest in the 12 months preceding the interview. Children who answered “No,” to the above question were placed in the “non-wheezing” group. Information was also collected regarding family history of allergy, SES, and environmental factors. Information on the participants’ history of pneumonia was retrieved from Matlab Hospital clinical records. Children with suspected pneumonia had been referred to Matlab Hospital from the CHRWs and the diagnosis of pneumonia was based on the WHO guidelines for the management of common illnesses with limited resources [26].

The procedures used for data collection in the 2001 study have also been previously described [9]. In 2001, Episodes of pneumonia was obtained from the RKS of Matlab HDSS. Pneumonia was recorded in the RKS by CHRWs during surveillance (performed every 2 weeks), based on statements from mothers regarding increased respiratory rates with or without chest indrawing.

### Numbers of death from respiratory infections including pneumonia and mid-year population

Information on deaths from respiratory infections including pneumonia among children under 5 years of age and the mid-year population in the area were obtained from HDSS Scientific Report Nos. 74, 82, 90, 103, 109, 121, and 138 published by the icddr,b [18–24]. The number of deaths from respiratory infections that include acute respiratory infection, pneumonia and influenza at < 1 year old and at 1–4 years old in the years 1991, 1996, 2001, 2006, 2009, 2011, and 2016, in the icddr,b service area of Matlab HDSS, as recorded in the Matlab HDSS Scientific Reports, were compared to find out the decrease in mortality. Since the 5-year-old participants of the 2001 study were < 1 year of age in 1996, and the 5-year-old participants of the 2016 study were < 1 year of age in 2011, the data for 1996 and 2011 are included here. Similarly, the data for 2006 and 2008 are included to compare the pre and post effect Hib combination vaccine on children of < 1 year of age and 1–4 years of age, respectively.

### Statistical analysis

This study was initially planned to determine the impact of the national deworming program on wheezing [9]. Therefore, the sample size was determined based on the levels of anti-*Ascaris* IgE. We calculated the sample size based on the assumption that at least 16% of children aged 60–71 months would have wheezing [27]. Given 80% power and a 5% significance level, 209 children in each group were required to detect a difference of 1.8 U_A_/mL (SD, 1.5) to 1.4 U_A_/mL (SD, 1.4) in the values of serum anti-*Ascaris* IgE levels between the wheezing and never-wheezing groups of children. Thus, we needed to recruit 240 children for the children with and without wheezing each, assuming a 15% refusal rate (including absences). To obtain the required number of children for the wheezing group, we needed to approach 1800 individuals, assuming a 20% loss due to absences, refusal, and other reasons.

Data were analyzed using IBM SPSS Statistics version 26 (IBM Japan, Tokyo, Japan). First, the prevalence of wheezing was calculated. An initial exploratory analysis was conducted to determine the distribution of the independent variables. After each variable had been subjected to a descriptive analysis between the children with and without wheezing, continuous variables (e.g., height) were compared using a *t*-test (if approximately normally distributed) or Mann–Whitney U test (if not normally distributed), and categorical variables were compared using a χ^2^ test. Then, the odds ratios for wheezing, with or without adjustments for risk factors, were calculated using multiple logistic regression analysis, with wheezing status as the outcome variable. Then, we compared the reduction in the history of pneumonia from 2001 to 2016 across for each age at the time of pneumonia history, and this reduction between 2001 and 2016 was analyzed using the paired *t*-test in both the overall study population and the wheezing group. We also compared the history of pneumonia between the 2001 and 2016 study participants for each age at the time of pneumonia development by using the χ^2^ test. We then defined the reduction rate of pneumonia (%) = (percentage of pneumonia cases in 2001 − percentage of pneumonia cases in 2016)/(percentage of pneumonia cases in 2001) times 100 (%), and made a graph to visualize it. The number of deaths from pneumonia among children aged 0 and 1–4 years during the period from 1991 to 2016 was analyzed using the χ^2^ test to determine the impact of various measures, such as antibiotic use by the CHRWs, Vitamin A distribution, improved hospital care, improved referral, improved nutritional status, including Hib combination vaccination, on mortality from respiratory infections including pneumonia in this area.

## Results

### Factors associated with wheezing

In the present study, the prevalence of wheezing during the 12 months prior to the survey was 8.7% (95% confidence interval [CI] 7.4%, 10.1%). The characteristics of the study participants are presented in Table 1. Wheezing children received significantly less Hib combination vaccination than children without wheezing, while antibiotic use during the first 1 year of life, a history of pneumonia at 1, 2, 3 and 4 years of age, parental asthma and allergic rhinitis were significantly more common in children with wheezing than in children without wheezing. Hib combination vaccination was a protective factor against wheezing (OR: 0.50; *p* = 0.001), while antibiotic use during the first 1 year of life, a history of pneumonia at 1 year of age (OR: 2.86, *p* = 0.046), at 2 years of age (OR: 3.18; *p* = 0.030), at 3–4 years of age (OR: 3.69; *p* = 0.030), and parental asthma were risk factors for wheezing (Table 2). Crude and adjusted odds ratios of pneumonia history at < 1 year were not significant for wheezing.

**Table 1:** Characteristics of the children by wheezing

		Wheezing	Non-wheezing	*p*
		(n = 145)	(n = 1513)	
Sex	M/F	73/72 (50.3%)	731/782 (48.3%)	0.640
Height	cm	107.0 ± 5.1	107.9 ± 5.4	0.073
Weight	kg	16.4 ± 2.7	16.5 ± 2.8	0.449
Hib vaccine^a^	Yes	125 (86.2%)	1397 (92.3%)	0.001
Deworming^b^	Yes	144 (99.3%)	1503 (99.3%)	0.968
Use of antibiotics^c^	Yes	138 (95.2%)	1315 (87.0%)	0.004
Contact with livestock^d^	Yes	72 (49.7%)	671 (44.4%)	0.220
Pneumonia history (years)^e^				
0	Yes	4 (2.8%)	32 (2.1%)	0.549
1	Yes	7 (4.8%)	17 (1.1%)	0.003
2	Yes	12 (8.3%)	18 (1.2%)	< 0.001
3–4	Yes	10 (6.9%)	14 (0.9%)	< 0.001
Mother has asthma	Yes	24 (16.6%)	105 (6.9%)	< 0.001
Mother has rhinitis	Yes	38 (26.4%)	289 (19.1%)	0.040
Father has asthma	Yes	19 (13.1%)	61 (4.0%)	< 0.001
Father has rhinitis	Yes	36 (24.8%)	200 (13.2%)	< 0.001
Monthly household income^f^	BDT^f^ median	10,000 (8000, 20,000)	12,000 (9000, 20,000)	0.228

^a^Record of Hib vaccine was drawn from the record of Matlab HDSS

^b^Has the child received any deworming drug?

^c^Did you give antibiotics during the first 12 months of your child’s life?

^d^Did the child have regular contact (at least once a week) with farm animals or livestock (e.g., cattle, goats, sheep, and poultry) during the first year of life?

^e^Data on pneumonia history were obtained from the clinical charts of Matlab Hospital, and pneumonia was diagnosed based on WHO criteria [20]

^f^Bangladesh Taka (1 Taka = $ 0.012)

**Table 2:** Risk factors for wheezing

		N	Crude OR^a^ (95% CI)	*p*	Adjusted OR (95% CI)^a^	*p*
Sex	M/F^b^	804/854	1.08 (0.77–1.53)	0.640	1.02 (0.71–1.45)	0.931
Use of antibiotics	Yes/no	1453/205	2.97 (1.37–6.44)	0.006	2.74 (1.24–6.06)	0.013
Hib vaccine	Yes/no	1522/136	0.52 (0.31–0.86)	0.011	0.50 (0.29–0.85)	0.010
Contact with livestock	Yes/no	743/915	1.24 (0.88–1.74)	0.220	1.28 (0.90–1.83)	0.174
Pneumonia at 0 years	Yes/no	36/1622	1.31 (0.46–3.77)	0.613	0.72 (0.23–2.30)	0.579
Pneumonia at 1 year	Yes/no	24/1634	4.46 (1.82–10.95)	0.001	2.86 (1.02–8.01)	0.046
Pneumonia a 2 years	Yes/no	30/1628	7.49 (3.53–15.89)	< 0.001	3.19 (1.12–9.08)	0.030
Pneumonia 3–4 years	Yes/no	24/1634	7.93 (3.46–18.20)	< 0.001	3.69 (1.13–12.06)	0.030
Mother has asthma	Yes/no	129/1529	2.66 (1.64–4.30)	< 0.001	2.58 (1.54–4.34)	< 0.001
Mother has rhinitis	Yes/no	327/1331	1.50 (1.02–2.23)	0.041	1.15 (0.74–1.79)	0.532
Father has asthma	Yes/no	80/1578	3.59 (2.08–6.20)	< 0.001	2.66 (1.46–4.84)	0.001
Father has rhinitis	Yes/no	236/1422	2.17 (1.45–3.25)	< 0.001	1.70 (1.08–2.67)	0.022

*OR* odds ratio, *CI* confidence interval

^a^Adjusted for each other

^b^Female is the reference category

### Pneumonia and wheezing

The history of pneumonia decreased as the children grew older in the 2001 study participants in both the overall cohort and the wheezing group. In contrast, it peaked at the age of 2 years in the 2016 wheezing group, whereas the history seems almost the same across the years in the 2016 overall cohort (Table 3).

**Table 3:** Number of the children with history of pneumonia in 2001 and 2016 study participants among the overall cohort and the wheezing group

Age of the pneumonia history	Number of children with a history of pneumonia n (%)		(χ^2^ test)
Overall cohort			
	2001 (n = 1580)	2016 (n = 1658)	*p*
0 years	287 (18.2%)	36 (2.2%)	< 0.001
1 year	185 (11.7%)	24 (1.4%)	< 0.001
2 years	101 (6.4%)	30 (1.8%)	< 0.001
3–4 years	30 (0.9%)	24 (0.7%)	0.196

Wheezing group			
	2001 (n = 219)	2016 (n = 145)	*p*
0 years	56 (25.6%)	4 (2.8%)	< 0.001
1 year	44 (20.1%)	7 (4.8%)	< 0.001
2 years	36 (16.4%)	12 (8.3%)	< 0.001
3–4 years	15 (3.4%)	10 (3.4%)	1.000

The pneumonia history significantly decreased from 2001 to 2016 in both the overall cohort and the wheezing group (paired t-test: *p* = 0.014 and *p* < 0.001, respectively). We found that in both the overall cohort and the wheezing group the pneumonia histories at the age of < 1 year, 1 year and 2 years significantly decreased in the 2016 (Fisher two-tailed exact test: *p* < 0.001), however, the pneumonia history did not significantly decrease at the age of 3–4 years in either of the overall study population or the wheezing group (*p* = 0.196 and 1.000, respectively) (Table 3).

### Deaths due to pneumonia

Table 4 shows that the percentage of deaths from respiratory infections including pneumonia continued to decrease significantly from 1996 to 2016 aged < 1 year (trend analysis: *p* < 0.001) and at the age 1–4 years (*p* < 0.001).

**Table 4:** Number of deaths from respiratory diseases including pneumonia and influenza

Year	0 years			1–4 years		
	Number of deaths from respiratory diseases	Mid-year population	Trend	Number of deaths from respiratory diseases	Mid-year population	Trend
1991	32 (1.18%)	2709	*p* < 0.001	11 (0.10%)^¶^	11,310	*p* < 0.001
1996	31 (1.26%)^†^	2457		5 (0.05%)^¶^	9772	
2001	29 (1.09%)^‡^	2660		2 (0.02%)^††^	9807	
2006	6 (0.23%)^‡^^§^	2583		1 (0.01%)	10,792	
2008	4 (0.15%)^||^	2642		2 (0.02%)	10,386	
2011	6 (0.25%)^†§||^	2433		1 (0.01%)	10,068	
2016	3 (0.11%)	2856		2 (0.02%)^††^	10,624	

^†^When compared between these years, when our study participants were at the age of < 1 year, the difference in the death rate at the age of < 1 year was significant (Fisher two-tailed exact test: *p* < 0.001)

^‡^When compared between these years, the differences in the death rate at the age of < 1 year was significant (Fisher two-tailed exact: *p* < 0.001)

^§^When compared between these years, before the implementation of Hib combination vaccination, the difference in the death rate at the age of < 1 year was not significant (Fisher two-tailed exact: *p* = 0.572)

^||^When compared between these years, before and after the implementation of Hib combination vaccination, the difference in the death rate at the age of < 1 year was not significant (Fisher two-tailed exact: *p* = 0.535)

^¶^When compared between these years, the differences in the death rate at 1–4 years was significant (Fisher two-tailed exact test: *p* < 0.001)

^††^When compared between these years, at the time of the 2001 and 2016 studies, the difference in the death rate in 1–4 years was not significant (Fisher two-tailed exact test: *p* = 0.292)

The percentages of deaths from respiratory infections including pneumonia aged < 1 year decreased significantly from 1996 to 2011, when our study participants were < 1 year of age (†: Fisher two-tailed exact test: *p* < 0.001), and from 2001 to 2006 (‡: Fisher two-tailed exact test: *p* < 0.001), however, it did not differ significantly when compared between 2006 and 2011 (§: Fisher two-tailed exact test: *p* = 0.572). Similarly, the decrease in the children aged < 1 year was not significant before and after the implementation of the vaccination (||: Fisher two-tailed exact test: *p* = 0.535). The deaths from respiratory infections including pneumonia at < 1 year old seems to have declined significantly in the year of 2006 in the area (data not shown).

In contrast, the difference at 1–4 years was significant between 1991 and 1996 (¶: Fisher two-tailed exact test: *p* < 0.001) but not significant between 2001 and 2016 (††: Fisher two-tailed exact test: *p* = 0.292), when our study participants of the 2001 and 2016 studies were1–4 years of age. The decline in the mortality among older children occurred in the early 1990′s earlier than the children aged < 1 year old.

### Reduction rates of pneumonia development

**Fig. 1:**
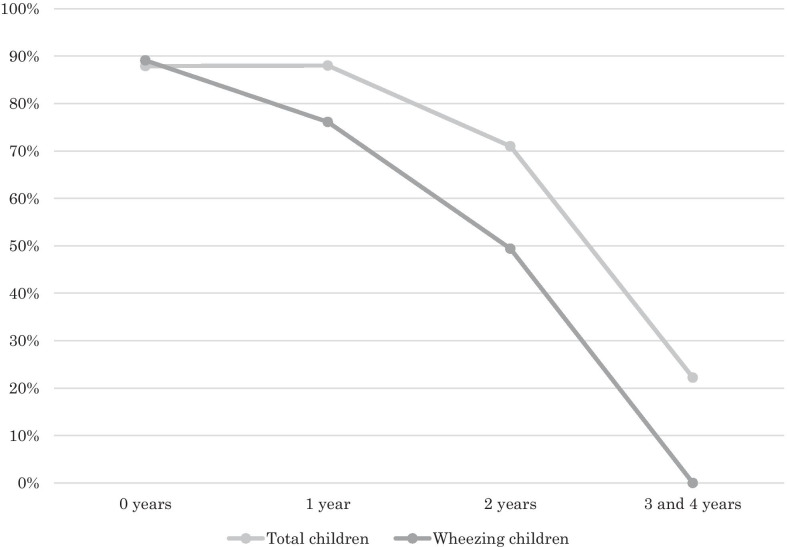
Reduction rate of pneumonia between the years 2001 and 2016 with ages in the overall cohort (total children) and the wheezing children. The light gray line of the polygonal line graph indicates the reduction rate of pneumonia between the years 2001 and 2016 in the overall cohort. The dark gray line of the graph indicates the reduction rate in the wheezing group. The pneumonia reduction rate decreased with age both in the overall cohort and the wheezing group, and it was more rapid in the wheezing group

The reduction rate of pneumonia defined by the following formula (%) = (percentage of pneumonia cases in 2001 − percentage of pneumonia cases in 2016)/(percentage of pneumonia cases in 2001) times 100 (%) at the ages of 0, 1, 2, and 3–4 years were 87.9%, 88.0%, 71.9%, and 22.2%, respectively, in the overall cohort, and 89.1%, 76.1%, 49.4%, and 0%, respectively, in the wheezing group (Fig. 1). Namely, whereas the reduction rate aged < 1 year was nearly 90%, the reduction rates aged 2–4 years were nearly less than 70%. It declined when the participants grew older and this decline was faster in the wheezing group.

## Discussion

The prevalence of wheezing was 8.7% in the 2016 study, which was significantly lower than that in the 2001 study. The Hib vaccine was found to be a protective factor against wheezing among 5-year-old children in rural Bangladesh. In contrast, a history of pneumonia at 1 to 4 years of age, antibiotic use in the first year of life, and parental asthma were risk factors for wheezing (Table 2). The history of pneumonia decreased as the children grew older in the 2001 study in both the overall cohort and the wheezing group (Table 3). In contrast, it peaked at the age of 2 years in the 2016 wheezing group, whereas it was almost the same in the 2016 overall cohort. The percentage of deaths from respiratory infections including pneumonia continued to decrease significantly from 1996 to 2016 aged < 1 year and at the age 1–4 years. The decrease was significant between 2001 and 2006 in the children aged < 1 year and between 1991 and 1996 in the children aged 1–4 years, however it was not significant in the children aged < 1 year between 2008 and 2011 just before and after the start of Hib combination vaccination, or between 2001 and 2016 in the children aged 1–4 years (Table 4). The pneumonia history significantly decreased from 2001 to 2016 in both the overall cohort and the wheezing group and the reduction rate in the history of pneumonia decreased as children grew older in both the overall cohort and the wheezing group, and it decreased more rapidly in the wheezing group (Fig. 1).

Whereas the participants of the 2001 study were most susceptible to pneumonia at the age of < 1 year, the 2016 wheezing group were most susceptible to pneumonia at the age of 2 years. Whereas the pneumonia reduction rate was around 90% aged < 1 year in both the overall cohort and the wheezing group, it decreased as the children grew older in both the overall cohort and the wheezing group and declined to less than 50% at the age 2 years (Fig. 1). The reduction in pneumonia development may be due to various measures which progressed during this time including Hib combination vaccination. Since other measures than Hib combination vaccination do not seem to have regressed during this period, the decline in the reduction rate may be attributable to Hib combination vaccination, which was started in 2009 before the participants of the 2016 study were born.

The effects of the Hib combination vaccination attenuate after 1 year or so when a booster dose is not administered [28]. The EPI of Bangladesh does not include a booster dose of the Hib combination vaccine, which likely resulted in the resurgence of pneumonia after 1 year of age (Tables 1 and 3). Table 2 shows the crude and adjusted ORs of the risk factors for wheezing. Pneumonia at the age of 1, 2 and 3–4 years was a risk factor for wheezing, whereas pneumonia at the age of < 1 year was not. We previously found that pneumonia history at the ages of 0, 1, and 2 years was a risk factor for wheezing [14, 27]. However, the present study did not show that a history of pneumonia at the age of < 1 year was a significant risk factor. This might be due to the prevention of pneumonia at the age of < 1 year by the Hib combination vaccination, whose efficiency then attenuated. These results are compatible with the fact that a booster dose of the Hib combination vaccine is not administered under the EPI in Bangladesh.

However, the reduction may be due to other measures, such as antibiotic use by the CHRWs, Vitamin A distribution, improved hospital care, improved referral, improved nutritional status, in the children aged < 1 year. In the Matlab HDSS service area, where our studies were conducted, the number of deaths from respiratory diseases including pneumonia of children aged < 1 year significantly decreased from 2001 to 2006 but no significant decrease was observed from 2008 to 2011, before and after the implementation of the Hib combination vaccination. Namely, Hib combination vaccination itself did not directly decrease the mortality from acute lower respiratory infections including pneumonia.

Among the measures taken, the effect on the reduction might have been attributable to Vitamin A distribution and improved nutritional status, since antibiotic use by CHRWs, improved hospital care and improved referral are related to the management of pneumonia and were unlikely to prevent pneumonia development. Figure 1 shows that the reduction rate of the development of pneumonia in the wheezing group declined faster than the overall cohort. It is unlikely that Vitamin A or nutritional improvements worked less effectively in the wheezing group, since they were not the risk factors for wheezing. Thus, the decline in the reduction rate in the wheezing group were more likely to be attributable to Hib combination vaccination rather than the other measures.

Three doses of Hib combination vaccine coverage in the Matlab HDSS service area has been more than 97% since the start of the vaccination [18–22] including the 2016 study participants (Table 1). DTP vaccination was widely in operation before introducing of the Hib combination vaccination with the three doses coverage more than 97% [23, 24] including the 2001 study participants (data not shown). Since hepatitis B does not seem to affect wheezing, these imply that the reduction in pneumonia history could be attributable to Hib vaccination.

Bronchial asthma in childhood is usually divided into three groups. One is called transient early wheezers who develop lower respiratory tract infections during young infancy but recover in later childhood. The second is atopic type with high IgE levels and the third is intermediate onset wheezers. Since children who experienced pneumonia have a higher risk of developing asthma in subsequent years, the symptoms of wheezing in the present study were compatible with these transient early wheezeres. The role of anti-Ascaris IgE antibody for the wheezy children is still necessary to be clarified [29].

Other risk factors for wheezing were antibiotic use during infancy, and maternal and paternal asthma. Antibiotic use during the 1st year of life has been reported to increase the rate of asthma, probably because it affects the normal development of the intestinal microbiome, especially among early wheezers, who develop wheezing after recurrent respiratory infections but grow out eventually [30–32]. The findings of this study are consistent with the above report. Parental asthma is an established predisposing factor for asthma, and the results of this study confirmed this.

The strength of this study lies in the method used for the selection of the study population. The participants were randomly selected from the general population of an area where strict registration of vital events has long been done, even though the study was conducted in a low- to middle-income country. Another strength of this study is the high response rate of 92.1%.

This study has certain limitations. First, different sampling methods were used. The 2001 study used random cluster sampling, while the 2016 study used random sampling [10]. Although the selection method differed, this guaranteed there was no selection bias in the two study populations. Second, the diagnostic criteria for pneumonia differed between the two studies [10]. Pneumonia was diagnosed based on the observed respiratory rate of the children by the guardians in the 2001 study, and this is regarded as the best method to detect pneumonia in a low-income country with limited resources. In the 2016 study, pneumonia was diagnosed by physicians according to the WHO guidelines, which were published after 2005 [21]. However, there was no significant difference in death rate between the years 2004 and 2007 (Fisher exact test; *p* = 0.584) [33, 34], which is before and after the introduction of the WHO guidelines respectively, indicating that the difference in the diagnostic criteria mattered little on mortality (data not shown). Third, in the 2001 study, the sample-size calculation was based on the prevalence of pneumonia among the population [10], while in the 2016 study, it was based on the difference in the serum IgE levels. Fourth, we used paired t-test to compare the difference in pneumonia development with age to assess the impact of Hib combination vaccine between 2001 and 2016. The ‘n’ should be at least 30 for a valid a paired t-test. However, the ‘n’ is essentially 4 (at 0, 1, 2 and 3–4 years) in the comparison. This indicates the weakness of this analysis.

## Conclusions

Hib combination vaccination shows a protective role against wheezing beyond target disease-specific protection at the age of < 1 year among 5-year-old children in rural Bangladesh, secondly due to the reduction of pneumonia. However, as a booster dose was not administered, this non-specific beneficial effect of this vaccination might have attenuated when the participants reached 1–4 years of age. An additional booster dose of Hib combination vaccine might further decrease the prevalence of childhood wheezing.

## Data Availability

Some of the datasets analyzed in the current study are available in the following Scientific Reports of the health and demographic surveillance system; Demographic Surveillance System-Matlab, v. 22. Registration of demographic events 1991, Scientific Report No. 74. Dhaka: ICDDR,B., Demographic Surveillance System-Matlab, v. 28. Registration of demographic events 1996, Scientific Report No. 82. Dhaka: ICDDR,B., Health and Demographic Surveillance System-Matlab, v. 34. Registration of health and demographic events 2001, Scientific Report No. 90. Dhaka: ICDDR,B., Health and Demographic Surveillance System-Matlab, v. 40. Registration of health and demographic events 2006, Scientific Report No. 103. Dhaka: icddr,b., Health and Demographic Surveillance System-Matlab, v. 42. Registration of health and demographic events 2008, Scientific Report No. 109. Dhaka: icddr,b., Health and Demographic Surveillance System-Matlab, v. 45. Registration of health and demographic events 2011, Scientific Report No. 121. Dhaka: icddr,b., Health and Demographic Surveillance System-Matlab, v. 51. Registration of health and demographic events 2016, Scientific Report No. 138. Dhaka: icddr,b., Demographic Surveillance System-Matlab, v. 37. Registration of demographic events 2004, Scientific Report No. 93. Dhaka: ICDDR,B. Available from: http://dspace.icddrb.org/jspui/handle/123456789/6494. Demographic Surveillance System-Matlab, v. 41. Registration of demographic events 2007, Scientific Report No. 106. Dhaka: ICDDR,B. Available from: http://dspace.icddrb.org/jspui/handle/123456789/6517. They are available from: http://dspace.icddrb.org/jspui/handle/123456789/6476 from: http://dspace.icddrb.org/jspui/handle/123456789/6514 from: http://dspace.icddrb.org/jspui/handle/123456789/6495 from: http://dspace.icddrb.org/jspui/bitstream/123456789/6500 from: http://dspace.icddrb.org/jspui/bitstream/123456789/6518 from: http://dspace.icddrb.org/jspui/handle/123456789/6521 from: http://dspace.icddrb.org/jspui/handle/123456789/9061 from: http://dspace.icddrb.org/jspui/handle/123456789/6494 from: http://dspace.icddrb.org/jspui/handle/123456789/6517 The other datasets generated and analyzed in the current study are not publicly available due to the restriction of the data only to the co-investigators of the study but are available from the corresponding author on reasonable request.

## Abbreviations

CI: Confidence interval
CHRW: Community health research workers
DTP: Diphtheria, tetanus, and pertussis
EPI: Expanded Programme on Immunization
HDSS: Health and demographic surveillance system
Hib: *Haemophilus influenzae* Type b
icddr,b: International Centre for Diarrhoeal Disease Research, Bangladesh [formerly known as ICDDR,B]
OR: Odds ratio
RKS: Record Keeping System
SES: Socio-economic status
WHO: World Health Organization
