# Correction to: Impact of *Haemophilus influenzae* type b combination vaccination on asthma symptoms and pneumonia in 5-year-old children in rural Bangladesh: a longitudinal study and comparison with a previous cross-sectional study

**DOI:** 10.1186/s12931-023-02502-6

**Published:** 2023-08-24

**Authors:** Haruko Takeuchi, S. M. Tafsir Hasan, Khalequ Zaman, Sayaka Takanashi, Samar Kumar Hore, Sultana Yeasmin, Shaikh Meshbahuddin Ahmad, Md Jahangir Alam, Masamine Jimba, Tsutomu Iwata, Md Alfazal Khan

**Affiliations:** 1 Department of Community and Global Health, Graduate School of Medicine, The University of Tokyo, 7-3-1 Hongo, Bunkyo City, Tokyo, 113-0033 Japan; 2 Nutrition and Clinical Services Division, International Centre for Diarrhoeal Disease Research, Bangladesh (icddr,b), 68, Shaheed Tajuddin Ahmed Sarani, Mohakhali, Dhaka 1212 Bangladesh; 3 Infectious Diseases Division, icddr,b, 68, Shaheed Tajuddin Ahmed Sarani, Mohakhali, Dhaka 1212 Bangladesh; 4 Department of Developmental Medical Sciences, Graduate School of Medicine, The University of Tokyo, 7-3-1 Hongo, Bunkyo City, Tokyo, 113-0033 Japan; 5 Infectious Disease Surveillance Center, National Institute of Infectious Diseases, 1-23-1 Toyama, Shinjuku City, Tokyo, 162-8640 Japan; 6 Organization for Population Health Environment & Nutrition, Adilpur Shastitala, Taltala Kheyaghat Road, Abhaynagar, Jashore, 7460 Bangladesh; 7 Department of Biochemistry and Microbiology, North South University, Bashundhara, Dhaka 1212 Bangladesh; 8 Department of Microbiology, Biomedicine Discovery Institute, Monash University, Clayton, VIC 3800 Australia; 9 The Graduate School of Humanities and Life Sciences, Tokyo Kasei University, 1-18-1 Kaga, Itabashi City, Tokyo, 173-8602 Japan

**Correction to: Takeuchi et al. Respir Res (2021) 22:35**

10.1186/s12931-021-01629-8

In the originally published version of the article [1], there were missing details in the Funding and Acknowledgements and should have been added.

In the Funding the information of and the International Community Health Research Fund was missing. In the Acknowledgements, the authors missed to express their thanks to for sincere and active involvement in the data collection of this study. The authors missed to express their thanks to Dr. Takahashi J, Dr. Yunus M, Dr. Chowdhury HR, Dr. Arifeen SE, Dr. Baqui A, Dr. Wakai S, Dr. Tariq Anwar, Dr. J. Chakraborty, Dr. Mahbubur Rahman, Professor David Sack, Dr. Yukiko Wagatsuma, and Professor Kanehisa Morimoto, for the active involvement in the creation of the dataset of the article with which this study was accomplished. They also missed erroneously to express their thanks to the study participants, the field research staff and Dr Ipshita Hamid Trisha who were sincerely involved in the data collection of the previous article (Reference 27). We have corrected the statement in the Acknowledgement. Below is the updated statement of the “Acknowledgement.”

## Funding

This work was funded by a Grant-in-Aid for Scientific Research (Basic Research (A), 26257507) from the Ministry of Education, Culture, Sports, Science and Technology, Japan, spanning the years 2014-2016, and the International Community Health Research Fund. It was also funded for unwavering support by the core donors of The International Centre for Diarrhoeal Disease Research, Bangladesh (icddr,b) - The governments of Bangladesh, Canada, Sweden, and the UK.

## Acknowledgements

The authors gratefully acknowledge all participating children who generously provided their invaluable blood and stool samples and their guardians who accompanied them. The authors gratefully acknowledge Prof. Shinji Nakahara also for statistical analysis. Moreover, the authors sincerely thank the field research staff and Dr. Ipsita Hamid Trisha for the rigorous data collection. The authors also acknowledge the valuable contributions of Dr. Junkichi Takahashi, Dr. Md Yunus, Dr. Hafizur Rahman Chowdhury, Dr. Shams EL Arifeen, Dr. Abdullah Baqui, and Susumu Wakai, who actively participated in creating the dataset referenced in this study (Reference 27), enabling its successful completion. Finally, the authors would like to express their appreciation to Prof. David Sack and Prof. Kanehisa Morimoto, Dr. Tariq Anwar, Dr. J. Chakraborty, Dr. Mahbubur Rahman, Dr. Yukiko Wagatsuma, for their kind assistance in supporting the creation of the dataset used in this study.

We apologize for the error.
